# Metastatic Colon Cancer in an 18-Year-Old without Predisposing Factors

**DOI:** 10.1155/2016/7820367

**Published:** 2016-03-27

**Authors:** Divya Mirchandani, Jolanta Kulpa, Nayaab Khawar, Israel Kochin, Pramod Narula, Revathy Sundaram

**Affiliations:** Department of Pediatrics, New York Methodist Hospital, Brooklyn, NY 11215, USA

## Abstract

While colorectal carcinoma is a common gastrointestinal cancer in adults, it is rare in pediatrics with an incidence of 1 : 1,000,000 and represents a fraction of neoplasms encountered in children. Malignant neoplasms represent a major cause of mortality in the pediatric age group. While presenting with weight loss, iron deficiency, rectal bleeding, abdominal pain, and change in bowel habits, or symptoms similar to acute appendicitis, the working diagnosis may be considered to be anorexia. This case illustrates the importance of considering colon cancer among other disease entities as a cause of unintentional weight loss in adolescents. While this is a rare occurrence in the pediatric population, significant unintentional weight loss with altered bowel habits should prompt a search for underlying malignancy—even in the absence of a positive family history or predisposing cancer syndromes.

## 1. Background 

Colorectal carcinoma is the most common type of gastrointestinal cancer in adults. It is a rare entity in pediatrics (<19 years) with an incidence of 1 : 1,000,000 and represents a fraction of neoplasms encountered in children [1, 2]. When leading causes of mortality are taken into consideration, malignant neoplasms represent the 4th overall cause of mortality in the 15- to 24-year age groups and 2nd in the 5- to 14-year age group [3]. Adequate statistical power remains a challenge in order to justify screening methods targeted at pediatric populations because of low incidence rates of 1.3 to 2 cases per million, as well as low 5-year survival rates ranging from 5% to 28% due to the aggressive features and advanced stage of the disease at primary diagnosis [4]. Colorectal cancer (CRC) generally presents with iron deficiency, rectal bleeding, abdominal pain, or change in bowel habits. Weight loss, anorexia, hepatomegaly, or ascites may also occur due to advanced disease. We report the case of a teenager with a presenting symptom of extreme weight loss who was found to have advanced liver metastasis and adenocarcinoma of the colon.

## 2. Case Report

An 18-year-old African American female was referred by her pediatrician to the emergency room for evaluation of significant weight loss (32 lbs) over a 3-month period. The past medical history was unremarkable. She had a good appetite and a well balanced diet. She stated that she experienced increased frequency of stools for 2 weeks prior to admission, nonmucoid and nonbloody in nature. There was no history of menorrhagia. The family history was negative for familial adenomatous polyposis (FAP).

Physical examination was normal except for tachycardia and acanthosis nigricans. Psychiatric evaluation was negative for depression or eating disorders. Further testing revealed hemoglobin of 9.7 g/dL consistent with iron deficiency anemia. The EKG revealed sinus tachycardia with a normal echocardiogram. The AST was 65 U/L and stool tested positive for occult blood. This prompted an abdominal ultrasound which unexpectedly revealed multiple hepatic lesions with the largest being 13 cm. The pelvic sonogram was negative. MRI of the abdomen and CT scan of the abdomen and pelvis confirmed these findings. Tumor markers were elevated with CEA of 5629 ng/mL and CA 19-9 of 1995 u/mL. She underwent a colonoscopy with biopsy. Colonoscopy revealed skip lesions and friable mucosa in the transverse colon. The pathology was consistent with moderately differentiated invasive adenocarcinoma of the transverse colon. She was referred to a cancer center for further treatment.

## 3. Discussion

Colon cancer is seen frequently in adults but rarely in children. We present the case of an 18-year-old female with weight loss who was initially thought to be anorexic. Psychiatric consultation ruled out eating disorders. Further evaluation led to a diagnosis of invasive adenocarcinoma of the colon with metastasis to the liver. This case illustrates the importance of considering colon cancer among other disease entities as a cause of unintentional weight loss in the pediatric population.

Adenocarcinoma of the colon in children is often seen in the setting of hereditary cancer syndromes which account for 5–10% of all cancers [5]. The incidence in the general population is 6% versus 60–80% in Lynch syndrome and >90% in FAP [6–8]. There is a significant association between acanthosis nigricans and gastrointestinal malignancy. A review of 277 cases shows 55% of acanthosis nigricans was associated with gastric carcinomas [9]. Since CRC in children and adolescents is rare the literature is limited to studies from single institutions [10, 11]. Recently a large population based study reviewed the occurrence of CRC in children and adolescents indicating that CRC has similar natural history as seen in adult patients [12]. Other studies signify that CRC is frequently overlooked in the differential diagnosis when children and adolescents present with symptoms including constipation, bloody stools, abdominal pain, and weight loss, whereas in adults the same history would prompt a colonoscopy [13–15]. Children and adolescents compared to adults are more likely to present with advanced stage of the disease at diagnosis, a higher occurrence of aggressive tumors, and poorer outcome [10–12, 16, 17]. Studies have shown that symptoms of CRC in pediatric patients were often present for several months (median of 3 months) before a diagnosis was made [10, 11]. Due to the rarity of the disease in the pediatric population, there is a delay in diagnosis and treatment [10–13, 16]. Whether the delay is due to the limited experience of pediatric oncologist or the aggressive behavior of disease in younger patients still needs to be explored [12]. Consultation with an oncologist experience in evaluating CRC in adults should be included. Efforts should be made to educate health providers in the early diagnosis of CRC in children and adolescents. Treatment for the pediatric population should be adapted from current adult treatment recommendations [10]. Participation in clinical trials addressing CRC would be advantageous although this is rarely available for the pediatric population. Chemotherapy has demonstrated a clear benefit for adults with stage III and IV disease and is actively evolving [13]. The FOLFOX regimen is the present treatment of choice [13]. Advancements in DNA sequencing and molecular biology should be explored in looking for potential biological markers of CRC that may reveal the differences between pediatric CRC and adult CRC [10].

## 4. Conclusion

Metastatic colon cancer in an 18-year-old female is a rare occurrence. Symptoms of CRC in pediatric and adolescent are often vague and nonspecific. In this case the presenting signs of significant unintentional weight loss, altered bowel habits, and anemia should prompt a search for underlying malignancy. Health providers should be educated on the early recognition of colorectal cancer even in the absence of a positive family history or predisposing cancer syndromes. We present this case to raise awareness in the early diagnosis and treatment of colorectal cancer in children.
